# Exploring Feature Selection with Deep Learning for Kidney Tissue Microarray Classification Using Infrared Spectral Imaging

**DOI:** 10.3390/bioengineering12040366

**Published:** 2025-03-31

**Authors:** Zachary Caterer, Jordan Langlois, Connor McKeown, Mikayla Hady, Samuel Stumo, Suman Setty, Michael Walsh, Rahul Gomes

**Affiliations:** 1 Interdisciplinary Quantitative Biology PhD Program, Biofrontier’s Institute, University of Colorado Boulder, Boulder, CO 80303, USA; ztcaterer@colorado.edu; 2 Department of Computer Science, University of Wisconsin Eau Claire, Eau Claire, WI 54701, USA; langloij0638@uwec.edu (J.L.); mckeowcr7239@uwec.edu (C.M.); 3 Department of Biology, University of Wisconsin Eau Claire, Eau Claire, WI 54701, USA; hadymp6527@uwec.edu; 4 Department of Neuroscience, University of Wisconsin Eau Claire, Eau Claire, WI 54701, USA; stumosm8453@uwec.edu; 5 Department of Pathology, University of Illinois Chicago, Chicago, IL 60612, USA; ssetty@uic.edu; 6 Biological Sciences Collegiate Division, University of Chicago, Chicago, IL 60637, USA; walshm@uchicago.edu

**Keywords:** laser-based infrared spectroscopic imaging, tissue microarrays, renal tumors, quantum cascade lasers, deep learning, feature selection

## Abstract

Kidney and renal pelvic cancer are a significant cause of cancer-related deaths, with the most common malignant kidney tumor being renal cell carcinoma (RCC). Chromophobe renal cell carcinoma is a rarer form of RCC that poses significant challenges to accurate diagnosis, as it shares many histologic features with Oncocytoma, a benign renal tumor. Biopsies for histopathological and immunohistochemical analysis have limitations in distinguishing chromophobe RCC from Oncocytoma. Syndromic cases may also have tumors with overlapping features. Techniques such as infrared (IR) spectroscopic imaging have shown promise as an alternative approach to tissue diagnostics. In this study, we propose a deep-learning-based framework for automating classification in kidney tumor tissue microarrays (TMAs) using an IR dataset. Feature selection algorithms reduce data dimensionality, followed by a deep learning classification approach. A classification accuracy of 91.3% was observed for validation data, even with the use of 13.6% of all wavelengths, thereby reducing training time by 21% compared to using the entire spectrum. Through the integration of scalable deep learning models coupled with feature selection, we have developed a classification pipeline with high predictive power, which could be integrated into a high-throughput real-time IR imaging system. This would create an advanced diagnostic tool for the detection and classification of renal tumors, namely chromophobe RCC and Oncocytoma. This may impact patient outcomes and treatment strategies.

## 1. Introduction

About 2.3% of all cancer deaths can be attributed to kidney and renal pelvic cancer. There were an estimated 13,920 deaths due to kidney and renal pelvic cancer in 2020, and about 79,000 more were diagnosed with this disease [1]. The most common type of renal malignancy is renal cell carcinoma (RCC), which accounts for about 90% of renal cancer diagnoses [2]. RCC has multiple histologic subtypes, of which the most prevalent form is clear cell RCC (ccRCC), followed by chromophobe RCC (ChRCC), accounting for 5–10% of renal malignancies. While ChRCCs are not associated with as high a mortality rate as ccRCC, they are of interest as they share similar morphological, histological, immunohistochemical, and ultrastructural characteristics with Oncocytoma, one of the most prevalent benign renal tumors [3,4]. A recent study using machine learning radiomics analysis indicates that computed tomography (CT) imaging may differentiate ChRCC from Oncocytomas [5]. Other studies indicate that this diagnostic approach may not be reliable on its own, as CT scans are non-specific [6].

Research suggests that early diagnosis of renal cancer can improve survival rates anywhere between 70–94% [7]. The need for a more reliable and efficient diagnosis of ChRCC has led researchers to explore alternative methods of diagnosis.

The current gold standard for the classification of kidney tumors is the use of histochemical stains such as Hematoxylin and Eosin (H&E) and colloidal iron to visualize the tissue and cellular morphology for patterns associated with renal tumors. This approach can be limited in its ability to differentiate between morphologically similar renal tumors, such as Oncocytoma and ChRCC. Several studies highlight the need for a novel biomarker solution for differentiating renal tumors [8]. Furthermore, a technique that can yield additional information about the chemical differences between tumor types would be advantageous. A promising method involves a label-free chemical imaging approach, such as infrared (IR) spectroscopic imaging, which can accomplish this objective by developing a pipeline capable of rapid detection and identification of biomarkers for these two diseases. IR imaging allows for the capture of images across more than just the visible wavelengths and can derive rich biochemical information from the tissues. Furthermore, developing a deep learning-based framework to automate this classification of renal tumors could potentially translate this process for clinical use. The intent is to significantly reduce the time of diagnosis and increase the reliability of prediction.

IR spectroscopy is an analytical method used to analyze molecules and their bonds by exposing the sample to the mid-infrared spectral range of 400–4000 cm^−1^ [9]. Exposure to the IR causes a dipole moment change which, when paired with an IR-sensitive detector, is used to obtain an IR absorbance spectrum, also termed a biochemical fingerprint [9]. This biochemical fingerprint can allow clinicians and researchers to implement this technique to identify diseased tissue. When interrogating biological material, the most important spectral regions are associated with the fingerprint region, which occurs between 900 and 1800 cm^−1^, respectively. The wavenumber region 2550–3500 cm^−1^ is associated with S-H, C-H, O-H, and N-H bonds and also can provide useful diagnostic information [9,10,11,12,13,14,15]. IR spectroscopy is a potential approach to interrogating kidney tissue such as cancer, diabetes, and fibrosis [16,17,18,19,20,21,22,23,24].

In this study, we develop a deep-learning framework that is capable of processing IR images and distinguishing between Oncocytoma and ChRCC. We explore the features of biomarkers related to the two tumor types. Deep learning algorithms have gained significant momentum due to their ability to accurately classify images by incorporating their spatial aspects. To enable this classification, we test deep learning models along with feature selection methodologies. Deep learning models with more hidden layers tend to have higher accuracy in image classification; however, adding more hidden layers increases model complexity, and the lack of publicly available data can make these deep learning networks challenging. We will use a framework that addresses data imbalance before training. This generates a lightweight deep learning model that can retain its predictive power while lowering the Floating-Point Operations Per Second (FLOPS) so that it can be integrated into a high-throughput real-time IR imaging system. The uniqueness of our study lies in integrating deep learning with Fourier Transform Infrared Spectroscopy (FT-IR) for renal cell carcinoma analysis while simultaneously addressing the challenges of low-resolution data and optimizing computational efficiency for real-world deployment.

## 2. Materials and Methods

### 2.1. Dataset

A tissue microarray (TMA) of formalin-fixed, paraffin-embedded renal tumors was created using tumors from the University of Illinois at Chicago Pathology archives (UIC IRB #2016-0581). Tissues were sectioned at 4 μm thickness with one section placed on a glass slide for histological staining and one section on a barium fluoride (${\mathrm{BaF}}_{2}$) slide for transmission IR imaging. The TMA consisted of 36 renal tumor tissue cores (1 mm diameter). Renal tissue from the cores had been identified as having 23 ChRCC tissue cores, 11 Oncocytoma tissue cores, one sarcomatoid RCC area core, and one papillary RCC core. The sarcomatoid and papillary RCC cores were excluded from the analysis. H&E staining was performed on adjacent tissue sections on a glass slide.

Three pathologists were involved in the review process to ensure diagnostic accuracy and minimize bias. The original sign-out pathologist established the initial diagnosis for patient care, utilizing clinical information, gross specimen features, and a comprehensive review of all glass slides, including H&E-stained, immunohistochemical, and special stains. The first study pathologist then reassessed the diagnosis, reviewing the report and slides to confirm the findings and selecting a representative tumor section. A second study pathologist further refined this selection by reviewing the chosen slide to create the tissue microarray. This multi-step process ensured that two pathologists independently confirmed the diagnosis before selecting the study sample, enhancing diagnostic robustness. Additionally, given the relative homogeneity of these tumors, selection bias was unlikely to significantly impact the study. The involvement of multiple pathologists strengthened the reliability of diagnosing chromophobe RCC and Oncocytoma. The ChRCC and Oncocytoma tumor types were determined for training and classification.

**Figure 1:**
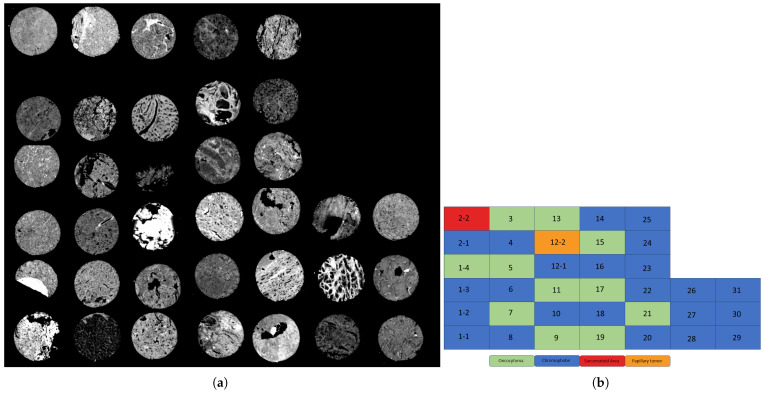
(**a**) Stitched Image of FT-IR of tissue microarray of ChRCC, Oncocytoma, Sarcomatoid RCC, and Papillary RCC. (**b**) shows Mapping of the ChRCC (blue), Oncocytoma (green), Sarcomatoid RCC (red), and Papillary RCC (orange) Data Set.

The tissues on the ${\mathrm{BaF}}_{2}$ slide were deparaffinized in hexane for 48 h before imaging, following established protocols. IR images were acquired with an Agilent Cary 600 series FT-IR spectrometer with a UMA 600 microscope from Agilent Technologies (Santa Clara, CA, USA), using the 15× objective with a 128 × 128 focal plane detector, at a pixel size of 5.5 μm × 5.5 μm, in transmission mode. A region of the slide that had been determined to be tissue-free was used to create a background image that was collected as a single tile before imaging. With a spectral resolution of 8 cm^−1^, 16 co-additions were acquired and averaged for each core in the TMA. Spectral images were acquired between 900 and 3850 cm^−1^. To remove spectral contributions from the substrate, atmosphere, and the global source, a background scan with 128 co-additions was carried out and ratioed to the single beam data [10]. Scattering through the sample was reduced by applying piecewise linear rubber band baseline correction [15]. This dataset is presented in Figure 1.

It is important to note that the renal tissues analyzed contain more than just tumor cells; as shown in Figure 1, the tissues appear to have multiple types within them, indicating heterogeneity. To ensure unbiased selection of Regions of Interest (ROIs), we consulted with the pathologist to gain a thorough understanding of RCC, including key cell types and distinguishing characteristics. Under the expertise of Dr. S. Setty, we manually selected ROIs to ensure that the point instances used in the analysis corresponded specifically to the tumor tissues. We also conducted a walkthrough of the ROI selection process using a traditionally stained biopsy sample (different from those included in this analysis) to refine our approach. This manual selection involved identifying and selecting point instances that represented each tumor type, excluding those associated with non-tumor-specific tissues such as background, cartilage, and connective tissue. We used the ROIs to extract all relevant point instances from the data for our analysis. The tumors were sampled from resections and studied as a TMA, where representative areas of the tumor were sampled. Therefore, the entire sample of specimens for each case was comprised of tumors, minimizing the need for exclusion beyond background areas. As the gold standard is the pathologists, this is an inherent limitation of these types of approaches.

### 2.2. Feature Selection

Discrete frequency infrared spectroscopy (DFIR) represents an approach for the collection of IR images only of the spectral frequencies of interest using a range of approaches. DFIR approaches allow for the selective targeting of specific frequencies of the IR spectrum rather than acquiring the entire mid-infrared spectral range by either using IR filters or by using single-wavelength lasers [25,26,27,28,29,30,31,32,33]. By collecting only spectral bands of interest, DFIR offers the advantage of reduced data acquisition times and lower computational demands, making it an appealing choice for high-throughput applications such as integration into the clinical setting [34]. While maintaining the ability to discriminate key spectral features, DFIR technology has demonstrated its potential for detecting diseases such as cancer through the analysis of serum and other biofluids [34,35,36,37,38,39,40,41,42,43,44].

With its capacity for real-time data collection, DFIR paves the way for the development of efficient and accurate diagnostic tools that could revolutionize healthcare by enabling earlier disease detection and intervention. Furthermore, DFIR lends itself to integration with newer IR imaging technologies using quantum cascade lasers (QCL) that allow for the collection of specific IR frequencies, which can lead to dramatic increases in data acquisition speeds and the potential for real-time imaging [34,35,36,37,38,39,40,41,42,43,44].

The multispectral imaging sensor can provide images with hundreds of spectral bands. Using fewer bands can drastically reduce computation time and resources; however, we need to verify from existing literature that the selected bands are indeed biologically relevant. With hundreds of bands produced in a single image, the hope is to reduce the total number of bands used in the training of the deep learning model down to a more manageable quantity to save time and resources by eliminating the need to run every single feature through our model. Feature selection algorithms were used to determine the statistically significant bands given in a particular scan that correlate to our regions of interest. Our approach takes three common feature selection algorithms: Extra Trees Classifier, Recursive Feature Elimination, and the ANOVA F–Test to create three separate feature importance rankings. This will be followed by a scheme to handle data imbalance issues before training. Data imbalance will be addressed by under-sampling the class with more data as well as exploring cross-validation schemes.

#### 2.2.1. Extremely Random Trees Classifier

The Extremely Random Trees Classifier, commonly referred to as the Extra Trees Classifier (ETC), is an ensemble learning method that combines bagging and random subspace methods. It is a variant of the Random Forest algorithm that differs in constructing individual decision trees within the ensemble. A random forest builds decision trees using a feature subset, which is created using the random subspace method. ETC takes this one step further by taking random thresholds for each feature instead of manually searching for a split point, thereby creating additional decision trees with randomized cut points. Although bootstrapped data sets are typically used for creating Random Forests, ETC uses the entire learning sample. Like any decision tree algorithm, the core of a pure split is measured using the GINI index, which is determined by the entropy values obtained from the features on which the split is made, as depicted in Equation (1).

(1) $$\mathrm{Gain}\left(S,A\right)=\mathrm{Entropy}\left(S\right)-\sum_{\nu \epsilon }\mathrm{Values}\left(A\right)\frac{\left|S_{\nu }\right|}{\left|S\right|}\mathrm{Entropy}\left(S_{\nu }\right)$$

ETC performs as accurately as, if not more accurately than, other ensemble methods. In addition to high accuracy, ETC also provides high computational efficiency [45]. ETC was used to aid in the feature selection of a machine learning algorithm that differentiates between one of four grades of Gastrodia elata [46]. ETC has also been applied to early-stage breast cancer diagnosis [46]. Researchers have utilized an extra-trees learning classifier in lung cancer identification using microRNA. The algorithm was determined to have 99.16% accuracy, 98.82% precision, and 98.88% recall [47]. ETC was used in tandem with Maximum Relevance—Minimum Redundancy to achieve 100% precision in a model that diagnosed early stages of liver disease [48]. However, this study has notable limitations and should warrant caution in interpreting and generalizing the findings.

#### 2.2.2. Recursive Feature Elimination

Recursive Feature Elimination (RFE) is an additional type of feature selection that eliminates weak features [49]. RFE reduces the number of features the core method uses to a preset number. It then ranks the features based on their importance. RFE uses an estimator to quickly narrow down and eliminate weak features from a given set. An estimator will train on the initial features and rank features based on its specific algorithm. As a smaller set is obtained, the RFE algorithm continues to call itself until a specified quantity of features is met for the final set. In our case, we used a Support Vector Machine (SVM) to implement the Linear Support Vector Regression (SVR) process. At each level of feature reduction, a simple linear regression model is fitted using SVR. Contrary to typical linear regression that tries to minimize the error between values, an SVR will attempt to fit a line between two support vectors based on a specific threshold. The change that an SVR makes to the typical linear regression line is shown in Equation (2), where |a| signifies the support vectors that the linear SVR attempts to satisfy. At each level of feature elimination, the feature furthest from the regression line will be eliminated.

(2) y−wx+b<|a|

RFE can increase accuracy and efficiency in random forest-based models [50]. A backward RFE algorithm was used in the feature selection of a machine learning model that predicted a prostate cancer patient’s fatigue level risk post-radiotherapy treatment [51]. RFE was used with a logistic regression model to diagnose breast cancer [52]. RFE was used as a form of feature selection in a model that achieved 99% accuracy in selecting genetically relevant genes in a leukemia gene expression dataset [53]. Alzheimer’s researchers found that a method using recursive feature elimination had a maximum of 90% accuracy in diagnosing Alzheimer’s from MRI scans [54].

#### 2.2.3. Analysis of Variance

Analysis of variance (ANOVA) is a method of comparing the variability of multiple distributions. By comparing the variances of different features, ANOVA can determine said feature’s importance. ANOVA examines the variability between distributions by computing the F-value, which is the ratio of two distributions divided by their degrees of freedom (DOF), as shown in Equation (3). The DOF for an ANOVA F-Test is a parameter that allows the user to set the maximum number of logically independent values. In practice, we used four degrees of freedom as an arbitrarily selected number. Once F-values are obtained for each feature, the *p*-value can also be calculated.

(3) $$F=\frac{\left(\frac{{\chi_{1}}^{2}}{n_{1}-1}\right)}{\left(\frac{{\chi_{2}}^{2}}{n_{2}-1}\right)}$$

ANOVA has been used as the primary source of feature selection in cancer testing by not only increasing computational efficiency but also reducing the cost of the test [55]. This method was also used in the feature selection of FT-IR spectroscopic scans of colon tissue to diagnose cancer [56]. ANOVA aided researchers in diagnosing COVID-19 from chest X-rays by “[reducing] computations and time complexity while overcoming the curse of dimensionality to improve accuracy” [57]. The model achieved 92% accuracy for multiclass classification and 98.72% accuracy for two-class classification [57]. A one-way ANOVA algorithm was used for feature selection in an SVM model that obtained 90% accuracy in selecting relevant DNA probes in microarray data of a leukemia dataset [58]. The highest accuracy (for an SVM) and lowest error for all models trained using the Wisconsin breast cancer dataset for breast cancer diagnosis were obtained by using an ANOVA-based model [59].

### 2.3. Deep Learning

Using the feature selection output, we tested the applicability of deep learning algorithms to predict renal tumors in kidney tissues. The deep learning model was tested in stages on the subsets of the data to identify which bands provide significant information for accurate classification. In total, 36 cores were used in the analysis. Of these, 4 cores came from one patient, and 2 cores came from two separate patients. However, one core from each patient was excluded from the analysis as it contained sarcomatoid RCC and papillary RCC, respectively, which were outside the scope of this study. The remaining 28 cores each came from unique patients, ensuring that no single patient’s data was overrepresented and minimizing the risk of overfitting.

A multilayer perceptron (MLP) is an artificial neural network that consists of fully connected layers of neurons. These layers include an input layer, multiple hidden layers, and an output layer. The neurons within the hidden and output layers contain an activation function, which returns zero if the weights are negative or the actual value if they are positive. Within the output layer, the sigmoid activation function is used. The Adam optimizer is also used, which is a stochastic gradient descent method utilizing the Adaptive Gradient algorithm (AdaGrad) and Root Mean Square Propagation (RMSProp) to dynamically adjust the learning rate for each parameter within the neural network. The binary cross-entropy loss function compares the predicted probabilities to the actual class label.

**Figure 2:**
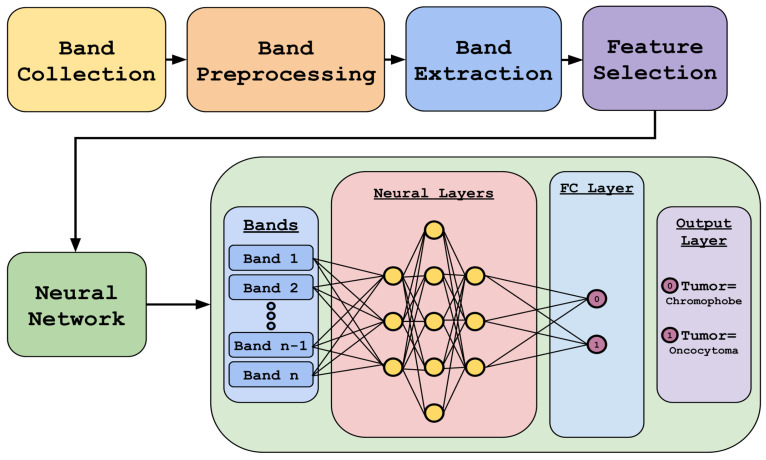
Overview of the entire training and validation process.

Figure 2 highlights our methodology. It includes data preprocessing and feature selection using different algorithms, followed by deep-learning-based classification using the reduced dataset.

The choice of an MLP over a Convolutional Neural Network (CNN) was primarily driven by our approach’s nature and the data’s characteristics. Our method was developed on a point-instance basis without assuming spatial locality, making an MLP a more suitable choice for this study. While CNNs are powerful for spatially structured data, they were not necessary for our current focus, which does not rely on spatial relationships. Additionally, there are two key reasons why spatially based approaches like CNNs would not be ideal for this specific dataset, including a limited spatial resolution where the IR imaging used in this study has a much lower spatial resolution than traditional visible or fluorescent microscopy. With a pixel size of 5.5 μm × 5.5 μm, the available spatial information (such as cell morphology) is inherently limited, reducing the advantage of CNNs. The second limitation is the TMA constraints, where the dataset consists of TMAs that sample multiple patients efficiently for biomarker discovery. However, these samples are small circular regions, providing minimal spatial context. This lack of spatial continuity makes CNNs less effective, as they typically leverage broader spatial relationships that are not well represented in this data structure. While CNNs have been used in our previous work [60], their applicability to this specific study is limited.

## 3. Results

Each feature selection model was imported using sci-kit-learn’s prebuilt algorithm and used for ranking features based on their importance. The goal was to reduce the original dataset into a much smaller subset to improve training speeds while hopefully producing similar results to the more intensive full dataset. Using multiple feature selection algorithms provides the advantage of pooling results, thereby extracting features that are reported to be consistently important for classification. In the initial phase, from the top 300 bands reported by each algorithm, a total of 104 bands were found to be common among them.

**Figure 3:**
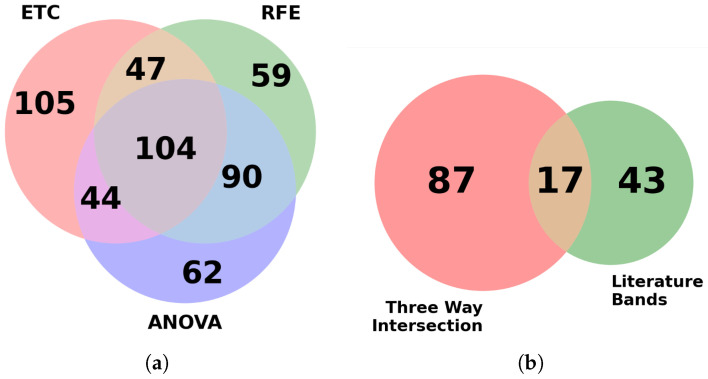
(**a**) Number of bands that were identified as important by the three feature selection algorithms. (**b**) Overlap between literature bands and the 104 bands common in (**a**).

**Figure 4:**
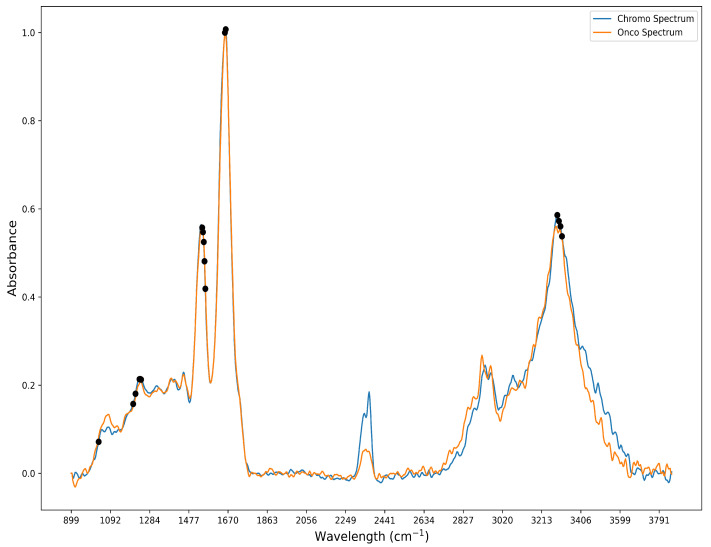
FT-IR spectra for ChRCC and Oncocytoma before band preprocessing. Black dots show an overlap between literature and feature selection for the 17 bands.

When attempting to cross-check our features against those known in the literature, a preliminary check of the top bands in our feature selection process was needed to see if there was a correlation. In the current standard, images are classified using a Bayesian classifier based on known biologically significant regions within the mid-infrared spectral range. To aid in the separation of spectral information among samples, four metrics are discussed in the literature review: peak-to-peak, peak-to-area, area-to-area, and area under the curve, which are employed. By comparing these metrics, which capture the unique spectral fingerprint of each biological sample, the metrics are ranked. As a result, 61 distinct spectral bands, previously validated by relevant research, are identified and utilized for spectral analysis of biological tissues [10,61,62]. These 61 bands were compared with our feature selection output for the detection of ChRCC and Oncocytoma. Further investigation revealed that 17 of these bands were also found in the 104 bands identified by the feature selection algorithm and shown in Figure 3. The 17 bands are predominantly localized to 4 main spectral peaks: amide I (1600–1700 cm^−1^), II (1500–1600 cm^−1^), III (1250–1350 cm^−1^), and A (3200–3300 cm^−1^), as shown in Figure 4 [10,63].

The neural network architecture was used to develop a model that performs binary classification into either Oncocytoma or ChRCC. These bands were carefully chosen based on the feature selection process. A total of 12 unique models were evaluated, which included different combinations of layer architectures and numbers of bands. Within the validation split, three different combinations of bands were used to train the model. The three different architectures differed in terms of the number of neurons present in each hidden layer. For example, one model had [100, 200, 300, 200] dense layers abbreviated as Model 1; the [50, 100, 150, 100] dense layers were known as Model 2; and [25, 50, 75, 50] were known as Model 3. The four different band configurations included the 767 bands model containing all bands, the 3-feature selection algorithm model containing 104 bands, the literature and feature selection intersection containing only 16 bands, and the literature bands containing 61 bands.

A total of 36,068 instances were extracted from the TMA using the ROIs and subsequently split into 70% for training and validation and 30% for testing on a random instance basis and selective core basis (Table 1). The 70:30 or 80:20 split for training/validation and testing is a common standard in AI/ML to ensure a balance between model training and evaluation. Since our dataset has a certain degree of imbalance, conforming to past research [64,65], a 70:30 split was chosen to ensure both classes had sufficient data for training while reserving an adequate portion for assessing model performance. A validation split of 30% was used to prevent overfitting. The batch size was set to 300, and the models were trained for 100 epochs. Table 2 explains the hyperparameters used for the deep learning model.

**Figure 5:**
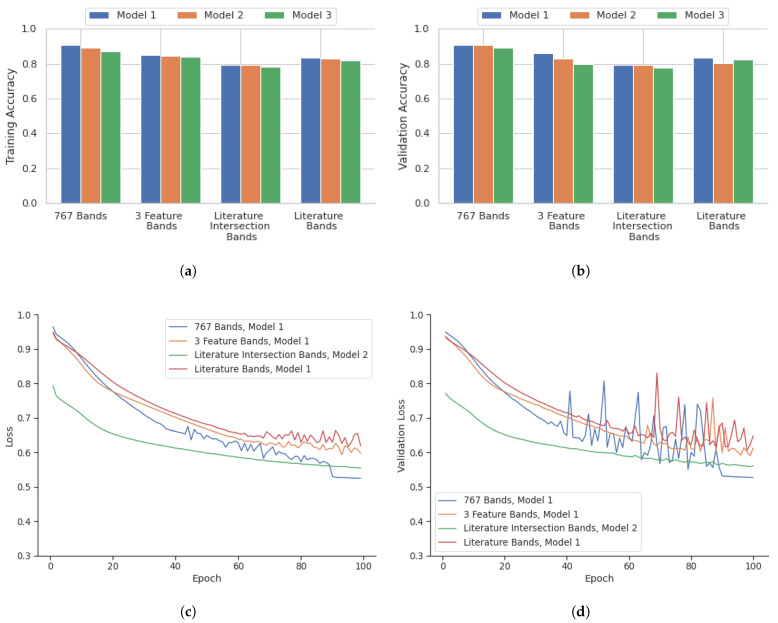
Overview of deep learning results on a point instance basis for three different architectures, Model 1, 2, and 3. (**a**) Training Accuracy and (**b**) Validation Accuracy, and deep learning training for 100 epochs of the best models as highlighted in the graphs: (**c**) Training Loss and (**d**) Validation Loss.

Figure 5 highlights the performance of the model in terms of accuracy, loss, accuracy of validation, and loss of validation for the analysis performed on a point-instance basis. After identifying the best deep learning models for the four datasets from the point instance, their performance was evaluated using the testing data. Table 3 shows the confusion matrix containing the outcomes.

**Figure 6:**
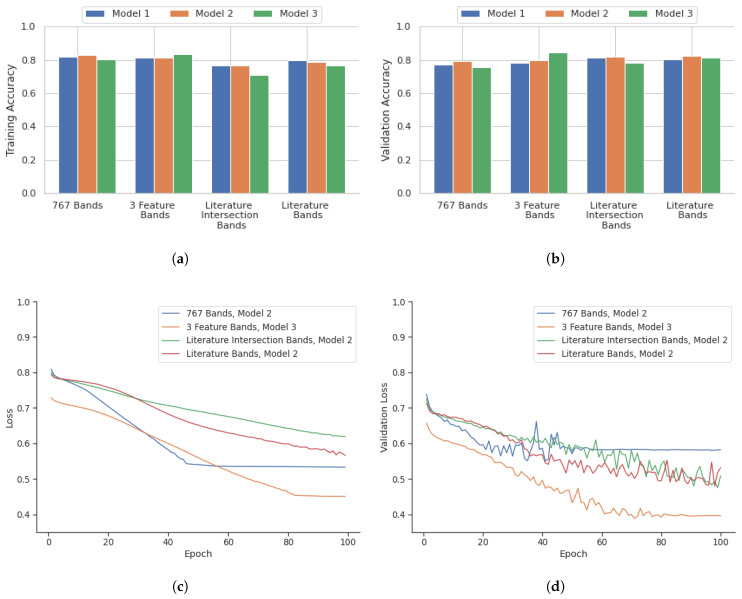
Overview of deep learning results on a core basis for three different architectures, Model 1, 2, and 3 (**a**) Training Accuracy and (**b**) Validation Accuracy, and deep learning training for 100 epochs of the best models as highlighted in the graphs (**c**) Training Loss and (**d**) Validation Loss.

A comparison of instance-based versus core-based analysis was also included in this research to identify potential areas of improvement. Figure 6 illustrates the performance of deep learning models over 100 epochs for core-based analysis in terms of accuracy, validation accuracy, loss, and validation loss. Similarly, the core-based analysis performance of the models was evaluated using the corresponding testing data. The confusion matrix containing the outcome for the core-based analysis is shown in Table 4.

## 4. Discussion

### 4.1. Point Instance Outcome

As highlighted in Figure 5, the 767 bands model showed the most consistent high accuracy and low loss values. This combination with Model 1 was the best performing out of all models evaluated, with a validation accuracy of 93.1%. The three feature selection algorithms model also showed slightly lower accuracy when compared to the 767 band model. The model with the highest performance with this combination was Model 1, with a validation accuracy of 85.2%. The literature and feature selection intersection bands model showed the lowest accuracy compared with all other band configuration models. Model 2 had the highest validation accuracy of 78.5%. The literature bands model had a comparable performance that was similar to the three-feature selection algorithm model. The best performing model for this combination was Model 1 with a validation accuracy of 83.0%. The training statistics for these best models over 100 epochs are shown in Figure 5.

Table 3’s confusion matrix containing the outcomes from the point instance performance on the testing data shows that 767 bands with Model 1 had 516 ChRCC and 419 Oncocytoma classes were misclassified, with 6564 ChRCC and 3250 Oncocytoma classes correctly classified (Table 3). The confusion matrix for Model 1 trained using the selected three feature bands had slightly more misclassified ChRCC classes at 1164 and 439 for Oncocytoma. The amount of correctly classified classes for ChRCC and Oncocytoma was 5916 and 3302, respectively (Table 3). The confusion matrix for bands identified from an intersection of literature and feature selection using Model 3 had the highest number of misclassified classes with 1407 ChRCC and 1046 Oncocytoma with 5673 ChRCC classes and 2695 Oncocytoma classes correctly classified (Table 3). Finally, the confusion matrix for literature bands using Model 1 showed only 1467 classes of ChRCC and 368 classes of Oncocytoma misclassified, while 5613 classes of ChRCC and 3373 classes of Oncocytoma were correctly classified (Table 3).

### 4.2. Core Based Outcome

The core-based outcome from Figure 6 illustrates that the 767-band and 3-feature band configurations displayed similar training accuracies, the highest being 82.9% for the 767-band Model 2 and 83.2% for the 3-feature band Model 3. However, the 3-feature band Model 3 outperformed in validation accuracy, achieving 84.3% compared to 79.1% for the 767-band Model 2. The literature intersection band selection exhibited the lowest training accuracy among all configurations, averaging 76. 9% for Model 2, but its validation accuracy improved to 82.0%. The configuration of the literature bands demonstrated consistency, with the highest training accuracy of 80.0% for Model 1 and a validation accuracy of 82.1% for Model 2. The training statistics for these models with the highest performance in 100 epochs are depicted in Figure 6.

The confusion matrix in Table 4 containing the outcome for the core-based analysis showed that for 767 bands with Model 3, 97 ChRCC and 2018 Oncocytoma classes were misclassified, while 7607 ChRCC and 2687 Oncocytoma classes were correctly classified (Table 4). The confusion matrix for Model 1, trained using the selected three feature bands, had slightly more misclassified ChRCC classes at 114 and 2242 for Oncocytoma. The number of correctly classified classes for ChRCC and Oncocytoma was 7590 and 2526, respectively (Table 4). The confusion matrix for bands identified from an intersection of literature and feature selection using Model 1 had the highest number of misclassified classes, with 296 ChRCC and 2607 Oncocytoma, and 7408 ChRCC classes and 2161 Oncocytoma classes correctly classified (Table 4). Finally, the confusion matrix for literature bands using Model 2 showed the best performance, with only 907 classes of ChRCC and 1390 classes of Oncocytoma misclassified, while 6797 classes of ChRCC and 3378 classes of Oncocytoma were correctly classified (Table 4).

The core-based analysis showed a decreased overall performance compared to the point-instance analysis. This comparison highlights that while point-instance-based analysis outperforms core-based analysis in terms of accuracy, the core-based analysis still shows a considerable degree of efficacy, particularly with the all-band configuration. Notably, the best-performing model and band configuration for core-based analysis (767 band Model 2 with a testing accuracy of 82.5% shown in Table 4) surpassed the worst-performing model and band configuration for point-based analysis (literature intersection with the highest testing accuracy of 78.5% for Model 2, shown in Table 3).

All bands identified from the literature review were considered in the feature selection process; however, only the overlap between the features from each selection algorithm was chosen. This method ensures that the selected features are the most robust and relevant for the specific classification task. Additionally, certain models with 61 literature-based features performed worse, likely because the number of features was insufficient to capture the complex relationships needed for optimal classification of RCC, rather than the selected bands being inherently poor performing. The selected 17 features represent a more refined and effective subset tailored to this specific application.

While the potential for overfitting is always a concern in machine learning models, we acknowledge that differences in performance between the point instance-based analysis (Table 3) and the core-based analysis (Table 4) suggest that patient-level overfitting may impact the results. Specifically, the higher sensitivity and specificity observed in the point instance-based analysis may reflect the lack of patient core screening, which could allow the model to learn patient-specific patterns rather than generalizable ones. In contrast, the core-based analysis, which excludes certain cores to avoid patient overfitting, exhibits lower overall specificity (around 50% or lower in some cases), raising concerns about the model’s robustness for a two-class prediction task. This observation highlights the challenges in developing a model that generalizes well to unseen data when patient-specific patterns are present. Moving forward, further optimization and a larger, more diverse dataset could help improve the model’s ability to balance sensitivity and specificity while avoiding patient-level overfitting.

Additionally, we have implemented regularization techniques, such as L1 and L2 regularization, to further mitigate the risk of overfitting by penalizing overly complex models. The learning rate was carefully selected to ensure efficient convergence without overshooting, and the Stochastic Gradient Descent optimizer was utilized for its effectiveness in balancing speed and accuracy. The point instance-based analysis, in particular, appears to provide more robust generalization by offering more diverse information for the model to learn from compared to the core-based analysis. Nevertheless, while the core-based approach shows similar training and testing accuracies, this reliability appears to be largely driven by the model’s high sensitivity rather than a balanced performance across both sensitivity and specificity. For instance, the discrepancy between sensitivity and specificity in Table 4 (e.g., 97% vs. 55%) suggests that the model is biased towards predicting ChRCC, which constitutes approximately 78% of all predictions. This imbalance raises concerns about overfitting to the dominant class, despite the close training and testing accuracies. Addressing this issue would require improving the model’s ability to distinguish between classes more effectively, particularly for the less frequent class, to ensure its broader applicability and robustness.

### 4.3. Model Efficiency

**Figure 7:**
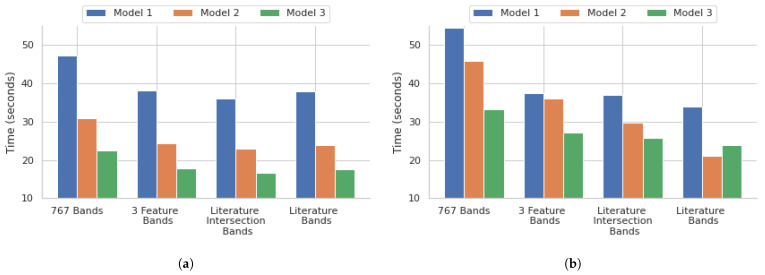
Time taken for training different deep learning architectures for the (**a**) point instance-based and (**b**) core-based analysis.

The performance of the different models demonstrated a few critical points within the scope of our research. The 3 feature selection algorithm bands with all the models showed that a significant reduction in the number of bands, down to those with the highest importance, shows a promising balance of both speed and accuracy. The reduction of the feature set down to 104, which is 13.56% of the original 767 bands, using the feature selection times of the 767 bands for all models, showed that they were reduced by approximately 21% for the 3 feature selection algorithm bands models, another key demonstration of the feasibility of this model. The literature bands, along with the literature and feature selection intersection bands for all models, had comparable training times to the 3 feature selection algorithm bands models, as shown in Figure 7. This shows that the adoption of DFIR-based approaches will predominantly enhance clinical relevance and translatability in addition to considerably reducing the number of resources that would be needed for band processing. The training didn’t further reduce the training times, but it did negatively affect the accuracy of the models.

## 5. Future Work

A need exists for differentiating ChRCC and Oncocytoma, as it is critical for accurate diagnosis in the clinical setting. This would lead to earlier intervention and improvements in patient outcomes. This study aimed to develop a classification approach for detecting these two types of tumors using IR spectral imaging coupled with deep learning models. Deep learning models with different architectures were evaluated and assessed using training and validation data. The results of this study showed that, depending on the band configurations, different models performed ideally when using the entire data set and with feature selection. Hence, the study showed that reducing the bands to those of the highest importance, evaluated by literature and/or computational approaches, significantly improved efficiency without compromising accuracy. Furthermore, the results showed the importance of careful selection of spectral bands and model architecture for optimal performance. These promising results lay the foundation for several potential future directions, such as the application of QCL laser-based imaging for clinical applications.

While spectral analysis is growing in classification and diagnosis settings, spectral information alone may not capture all necessary information for accurate classification and detection. Integrating additional data sources, such as genetic or proteomic data, could enhance the classification’s performance and provide a more comprehensive understanding of the underlying biology. Additionally, while this study employed deep learning models for classification, architectures that are specifically designed for analyzing spatial/spectral information could be investigated to improve the model’s performance, such as CNNs or recurrent neural networks. While these additional architectures may provide novel information, it is critical to validate the developed models on independent datasets to assess their construction. To validate and improve our model, we plan to implement various methods, such as cross-validation, bootstrapping, and other robust statistical techniques to ensure its reliability and generalizability. Alternative methods, such as oversampling, could be explored in future work to assess their impact on model performance. It would be interesting to observe if the randomness of tree-based algorithms can be reduced by different oversampling techniques.

It is also important to bridge the gap between classification and detection. While classification is important and can complement detection, detection allows us to identify and locate specific objects within a dataset, enabling us to gain insights, make informed decisions, and take appropriate actions based on the detected instances. Coupling-optimized classification and detection models will be explored to evaluate their clinical performance. Conducting studies involving clinical setting patient samples in assessing the model’s performance would be an important future direction, particularly in developing a classifier on a single TMA that captures traditional clinical variations without the need for manual selection of ROIs.

Several barriers exist to integrating IR imaging into clinical practice, including meeting medical standards, addressing liability concerns (especially regarding false positives and negatives), and ensuring compatibility with existing diagnostic workflows. Training requirements for clinical staff, time constraints, and resistance to changing established pathology practices also pose challenges. Additionally, high equipment costs, ongoing maintenance, and limited access to large patient datasets, particularly for rare diseases, could hinder adoption. Further obstacles include a lack of standardization across IR spectroscopy instruments and research labs. However, initiatives like ClirSpec are actively working to overcome these barriers.

This study has shown the utility of IR spectral information on chromophobe Renal Cell Carcinoma and Oncocytoma tissue samples. Spectral information can be collected across various forms of biological tissue. Future research could benefit from expanding the dataset to include additional types of renal tumors or other types of cancers. This would provide a more comprehensive analysis and potentially improve the model’s performance on different tumor types, or may allow for the optimization of the model architecture depending on the tumor type.

## Figures and Tables

**Table 1:** Training, Validation, and Testing Split from Dataset on a point and core basis.

Instance	Cancer	Cores	Total Classes	Training Classes	Validation Classes	Testing Classes
Point	ChRCC	23	23,599	11,564	4955	7080
	Oncocytoma	11	12,469	6110	2618	3741
	Total	34	36,068	17,674	7573	10821
Core	ChRCC	23	23,599	11,126	4769	7704
	Oncocytoma	11	12,469	5391	2310	4768
	Total	34	36,068	16,517	7079	12,472

**Table 2:** Deep Learning model parameters and values for three model variations used in the experiments.

Parameters	Model 1	Model 2	Model 3
# Neurons in Hidden Layers	100, 200, 300, 200	50, 100, 150, 100	25, 50, 75, 50
Epochs	100		
Optimizer	Stochastic Gradient Descent		
Loss	Binary Crossentropy		
Batch Size	300		
Learnable Params	217,701	73,851	28,176
Learning Rate (LR)	max = 0.01 and min = 0.0001		
LR Patience and Reduction	10 and 0.1		
Regularizer	L1 (1 $\times \;10^{-5}$) and L2 (1 $\times \;10^{-4}$)		
Activation	Relu and Sigmoid (last layer)		

**Table 3:** Comparative performance analysis of models 1, 2, and 3 across different testing datasets (767, 3 Feature, Literature, Literature Intersection) using confusion matrices to evaluate sensitivity (s), specificity (p), and accuracy on a point instance basis.

Dataset	Model	Actual	Predicted		Predictive Values	Accuracy
767			ChRCC	Onco		
	Model 1	ChRCC	6564	516	s: 92.7%	91.3%
		Onco	419	3250	p: 88.6%	
	Model 2	ChRCC	6565	515	s: 92.7%	90.5%
		Onco	508	3233	p: 86.4%	
	Model 3	ChRCC	6524	556	s: 92.1%	88.9%
		Onco	647	3094	p: 82.7%	
3 Feature	Model 1	ChRCC	5916	1164	s: 83.6%	85.2%
		Onco	439	3302	p: 88.3%	
	Model 2	ChRCC	5446	1634	s: 76.9%	82.2%
		Onco	288	3453	p: 92.3%	
	Model 3	ChRCC	5110	1970	s: 72.2%	79.6%
		Onco	238	3503	p: 93.6%	
Literature	Model 1	ChRCC	5613	1467	s: 79.3%	83.0%
		Onco	368	3373	p: 90.2%	
	Model 2	ChRCC	6732	348	s: 95.1%	79.9%
		Onco	1822	1919	p: 51.3%	
	Model 3	ChRCC	6572	508	s: 92.8%	81.6%
		Onco	1478	2263	p: 60.5%	
Literature Intersection	Model 1	ChRCC	5913	1167	s: 83.5%	78.2%
		Onco	1187	2554	p: 68.3%	
	Model 2	ChRCC	5949	1131	s: 84.0%	78.5%
		Onco	1200	2541	p: 67.9%	
	Model 3	ChRCC	5673	1407	s: 80.1%	77.3%
		Onco	1046	2695	p: 72.0%	

**Table 4:** Comparative performance analysis of models 1, 2, and 3 across different testing datasets (767, 3 Feature, Literature, Literature Intersection) using confusion matrices to evaluate sensitivity (s), specificity (p), and accuracy in a core-based analysis.

Dataset	Model	Actual	Predicted		Predictive Values	Accuracy
767			ChRCC	Onco		
	Model 1	ChRCC	7482	222	s: 97.1%	81.2%
		Onco	2118	2650	p: 55.6%	
	Model 2	ChRCC	7275	429	s: 94.4%	80.8%
		Onco	1962	2806	p: 58.9%	
	Model 3	ChRCC	7607	97	s: 98.7%	82.5%
		Onco	2081	2687	p: 56.4%	
3 Feature	Model 1	ChRCC	7590	114	s: 98.5%	81.1%
		Onco	2242	2526	p: 53.0%	
	Model 2	ChRCC	7581	123	s: 98.4%	80.0%
		Onco	2370	2398	p: 50.3%	
	Model 3	ChRCC	7301	403	s: 94.8%	78.9%
		Onco	2228	2540	p: 53.3%	
Literature	Model 1	ChRCC	6773	931	s: 87.9%	81.4%
		Onco	1393	3375	p: 70.8%	
	Model 2	ChRCC	6797	907	s: 88.2%	81.6%
		Onco	1390	3378	p: 70.8%	
	Model 3	ChRCC	7664	40	s: 99.5%	80.3%
		Onco	2412	2356	p: 49.4%	
Literature Intersection	Model 1	ChRCC	7408	296	s: 96.2%	76.7%
		Onco	2607	2161	p: 45.3%	
	Model 2	ChRCC	7161	543	s: 93.0%	78.1%
		Onco	2184	2584	p: 54.2%	
	Model 3	ChRCC	7634	70	s: 99.1%	81.9%
		Onco	2183	2585	p: 54.2%	

## Data Availability

The datasets presented in this article are not readily available because IRB #2016-0581. Requests to access the datasets should be directed to walshm@uchicago.edu. All code can be found at https://github.com/caterer-z-t/AI-Bioimaging (accessed on 10 June 2024).
